# Morphology and gonad development of normal soldiers and reproductive soldiers of the termite *Zootermopsis nevadensis nevadensis* (Isoptera, Archotermopsidae)

**DOI:** 10.3897/zookeys.148.1672

**Published:** 2011-11-21

**Authors:** Susan E. Johnson, Nancy L. Breisch, Bahram Momen, Barbara L. Thorne

**Affiliations:** 1 Department of Entomology, University of Maryland, College Park, MD 20742, USA; 2 Department of Environmental Science & Technology, University of Maryland, College Park, MD 20742, USA

**Keywords:** evolution of soldier caste, reproductive soldier, neotenic soldier, *Zootermopsis*, morphometrics

## Abstract

Reproductive or neotenic soldiers of the Archotermopsid *Zootermopsis nevadensis* *nevadensis* (Hagen) are compared to sterile soldiers and primary male reproductives. Several head capsule morphometrics correlate significantly with gonad size across all forms and both sexes of soldiers. The easily observed field character of ratio of mandible length to labrum length is a consistent and reliable feature of head capsule external morphology for predicting gonad development and reproductive potential of soldier forms regardless of age, sex, or live weight.

## Introduction

Soldiers are a non-reproductive defensive caste in termites (though they may sometimes have other roles (Traniello 1981) and are not always the exclusive defensive caste in a colony (e.g. Nel 1968, Thorne 1982, Haverty and Thorne 1989, Polizzi and Forschler 1998, Delphia et al. 2003)). Compared to other colony members they generally have large, heavily sclerotized heads with enlarged mandibles, although some derived groups (e.g. *Nasutitermes*) have vestigial mandibles and rely on chemical defense (reviewed by Weesner 1969a). A soldier caste is found in all termites except in the Termitid subfamily Apicotermitinae, in which they have been secondarily lost (reviewed in Inward et al. 2007). Primitive termites of the family Archotermopsidae (Engel et al. 2009) retain developmental plasticity which allows all castes (except normal soldiers) to become reproductive, either through development into an alate (winged dispersal form) or through molts into a non-dispersive *neotenic* (replacement) reproductive (Light and Weesner 1951, Noirot 1985, Noirot and Pasteels 1987, Thorne 1996, Roisin 2000). Although they retain the prothoracic glands necessary for molting, soldiers are the exception to the Archotermopsids’ overall developmental flexibility, and are considered a terminal caste because they do not molt again (Noirot and Pasteels 1987). However, in six species of primitive termites, (Archotermopsidae: *Archotermopsis wroughtoni* Desneux, *Zootermopsis angusticollis* (Hagen), *Zootermopsis nevadensis* (Hagen)*, Zootermopsis laticeps* (Banks); Stolotermitidae: *Stolotermes brunneicornis* (Hagen), *Stolotermes ruficeps* Brauer), some neotenics of both sexes have soldier-like morphological characteristics, and are called *reproductive soldiers*, or *neotenic soldiers* (reviewed by Myles 1986). These soldier-like neotenics are phylogenetically rare and have been reported only occasionally in most of the six species, but are found more commonly in *Archotermopsis wroughtoni* and *Zootermopsis nevadensis* (Imms 1919, Thorne et al. 2003). Reproductive soldiers possess large mandibles similar to those of normal soldiers but have fully developed gonads upon sexual maturity (Imms 1919, Heath 1928, Castle 1934, Myles 1986). *Zootermopsis nevadensis* reproductive soldiers behave more like reproductives than soldiers (Heath 1928, Thorne et al. 2003).

Because reproductive soldiers occur only in the most socially and developmentally primitive termites, they are considered probable evolutionary relicts of an early form of soldiers: a stepping-stone toward obligatory sterility and altruistic defense (Thorne et al. 2003).

Reproductive soldiers, while possessing the generalized soldier form, typically have differences in external morphology that distinguish them from normal soldiers including a slightly rounder head shape and more curved mandibles (Heath 1928, Myles 1986). The abdomen may appear banded due to expansion of the intersegmental membrane between sternites as well as an increase in fat body and changes in its distribution (N. Breisch 2011 pers. obs.) and consistency, possibly royal fat body (Grassé 1982). However, there is often individual variation in external morphology (Light 1943, Morgan 1959, Thorne et al. 2002, Thorne et al. 2003).

Here we compare external morphology and internal gonad development in normal and neotenic soldiers of *Zootermopsis nevadensis nevadensis* (Thorne & Haverty, 1989) and in new kings and mature kings. Using measurements of several external features as well as gonad dimensions, differences are quantified between normal soldiers and reproductive soldiers (referred to collectively henceforth as “soldier morphs”). Ratios of these measurements (used to normalize the expected differences due to size and age of individuals) are analyzed for predictive value (Light 1927). Gonad size is correlated with age and live weight in male soldier morphs and primary male reproductives (kings).

## Methods

### Experimental production of replacement reproductives

Kings were removed from 64 king and queen right colonies to stimulate production of male replacement reproductives. Colonies were outbred, initiated by alate pairs that emerged from wild colonies collected near Placerville, CA (El Dorado County). At 2 wk intervals beginning 6 wks post king removal colonies were examined for replacement (neotenic) reproductive soldiers. Twelve new male reproductive soldiers were weighed live then individually preserved in Pampel’s fixative (composed of 2 - 4 parts glacial acetic acid , 15 parts 95% ethyl alcohol, 30 parts distilled water, 6 parts formalin (40% formaldehyde in water): BioQuip Products). Ten mature (molted to soldier morph at least 3 months previously) male reproductive soldiers and ten mature normal/sterile (molted to soldier morph at least 3 months previously) male soldiers from similar sized colonies were also weighed live and individually preserved. After 24 h in fixative external characters (width of head capsule, length of head capsule, length of left mandible from condyle to apex, length of labrum, width of labrum) of specimens were measured using an eyepiece-mounted micrometer on a Leica MZ MPO dissecting microscope. Each termite was then pinned to a paraffin-filled Petri dish and a longitudinal incision was made on the dorsal side. The open body cavity was flooded with a solution of Nile Blue dye and water then rinsed with 70% ethanol after several seconds leaving enough ethanol to partially cover the specimen. Widest and narrowest diameter of the left testis was measured. Testis width subsequently refers to the widest measurement of the left testis for specimens of known age and weight.

### Characterization of dealate and mature male primary reproductives

Newly sclerotized male alates (new kings) were individually isolated with a 2 cm square of moistened paper towel until they shed their wings. After wing abscission they were weighed, preserved in Pampel’s fixative and analyzed as above. A subsample of the kings removed from colonies to generate secondary reproductives were also weighed, preserved and analyzed as above. All these kings were at least two years old.

### Characterization of archived specimens of reproductive and normal soldiers

In addition to the production of known age reproductive soldiers, previously collected individuals were classified as “normal soldiers” (n = 144; 84 male, 60 female) or “reproductive soldiers” (n = 47; 38 male, 9 female) based upon external morphology and observed colony role. The majority (192) were from outcrossed laboratory colonies, which were bred from alates maturing in colonies initiated by alate pairs that emerged from wild colonies near Placerville, CA (El Dorado County). Ten individuals developed in and were collected directly from the field-collected stock colonies. Sixteen were collected directly from the field in October 2007 from Eldorado National Forest (El Dorado County, CA). The field collected individuals were preserved in ethanol without fixative, 79 were fixed in Bouin’s solution (composed of 37% formaldehyde (24% by weight), picric acid (71%), and glacial acetic acid (5%): BBC Biochemical Corporation) for at least an hour before transfer to 80% ethanol, and 115 were fixed and stored in Pampel’s. Eight had previously been preserved in an unidentified fixative (ethanol and/or Pampel’s).

The external measurements of each soldier morph individual included: dorsal width of head capsule at widest point, dorsal length of head capsule without mandibles from the posterior margin to the base of the labrum, length of left mandible from condyle to apex, length of labrum, width of labrum, wingbud length (if present), width of postmentum at narrowest point, width of postmentum at widest point, length of postmentum, length of eye, and width of eye. Sex was recorded as well. After external measurements were completed the following measurements were taken: females—width of ovary at midpoint, width of ovary at widest point, length of ovary from tip to base of posterior ovariole, number of eggs; males—widest diameter of testis, narrowest diameter of testis. Ratios of head, labrum, and testis lengths and widths were made for each individual as a measurement of roundness. The ratio of the mandible length to the labrum length was also calculated. These morphometrics were suggested as useful differentiating characteristics for soldier morphs by laboratory experience and the published anatomical work on the reproductive system of *Zootermopsis nevadensis* *nevadensis* and other termites by Light (1927), Child (1934) and Weesner (1969b).

## Analysis

All data were analyzed using SAS 9.1 for Windows (Correlation Analysis, MANOVA, ANOVA). Results were considered significant at the 0.05 level. Correlations were univariate, and thus may result in an overall type I error rate greater than the pair-wise rate of 0.05.

## Results

### Specimens of known age and live weight

***Contrasting live weight of normal soldiers to reproductive soldiers and kings***

**Figure 1.**
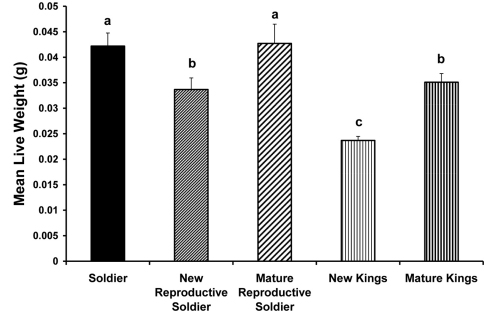
Comparison of means for live weight (g) of male soldiers, reproductive soldiers and primary reproductives (new and mature kings). Means with the same letter are not significantly different. Error bars indicate standard errors.

Newly differentiated male reproductive soldiers were smaller (mg live weight) than either normal sterile soldiers or mature reproductive soldiers (p < 0.0001) but not different from mature kings. Normal sterile soldiers and mature reproductive soldiers did not differ in weight. Dealate kings were smaller than the other four morphs/castes (p < 0.0001) (Fig. 1).

***Contrasting external morphology and gonad size of normal and reproductive soldiers***

**Figure 2.**
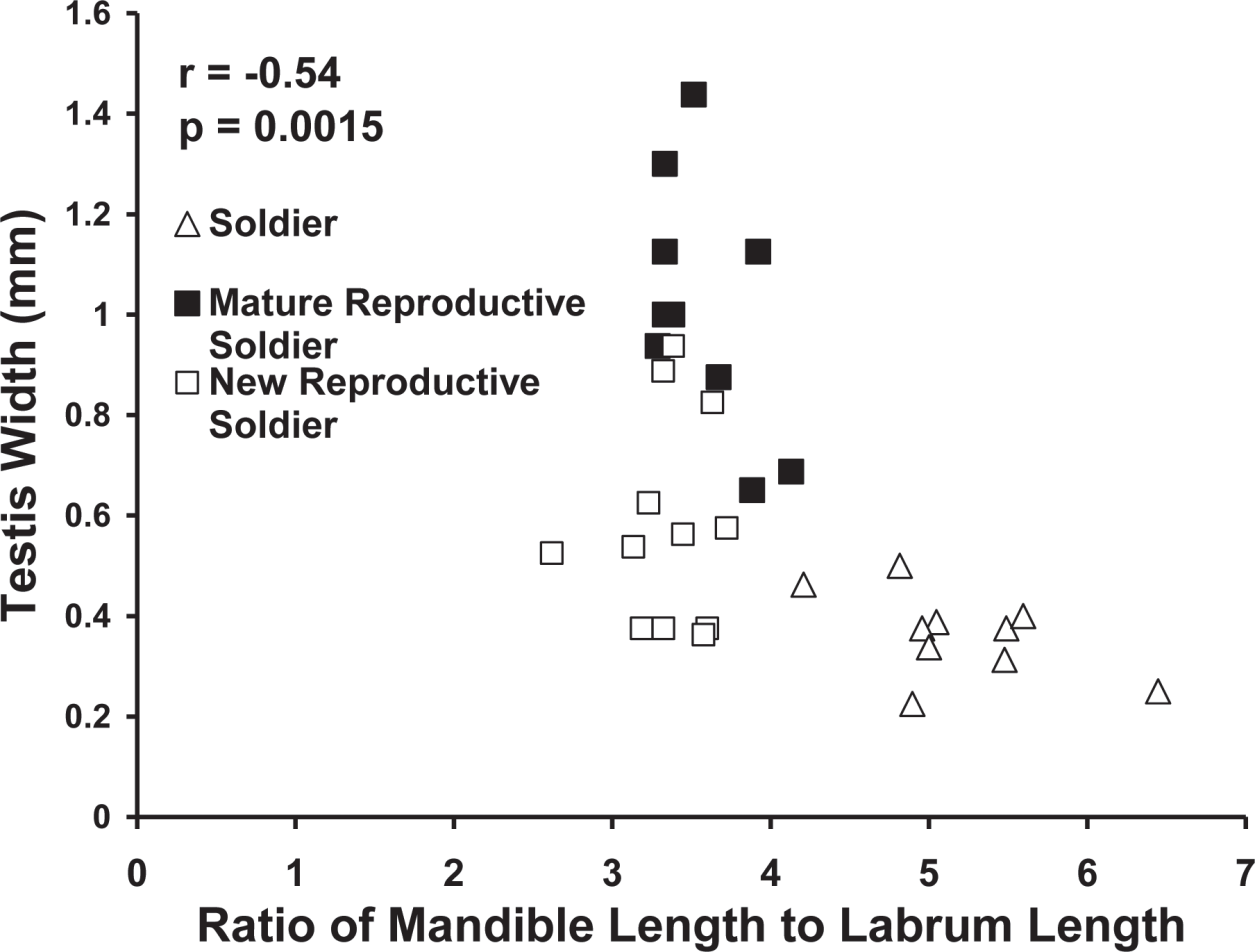
Correlation between testis width and ratio of mandible length to labrum length in male soldiers and new and mature reproductive soldiers.

The ratio of labrum length to left mandible length distinguished sterile from reproductive soldier morphs (p < 0.0001). Reproductive soldiers had larger testes than sterile soldiers regardless of live weight or age of reproductive soldier (p < 0.0001). Testis width correlated with the ratio of left mandible length to labrum length (p = 0.0015) (Fig. 2).

***Age effects on gonad development***

**Figure 3.**
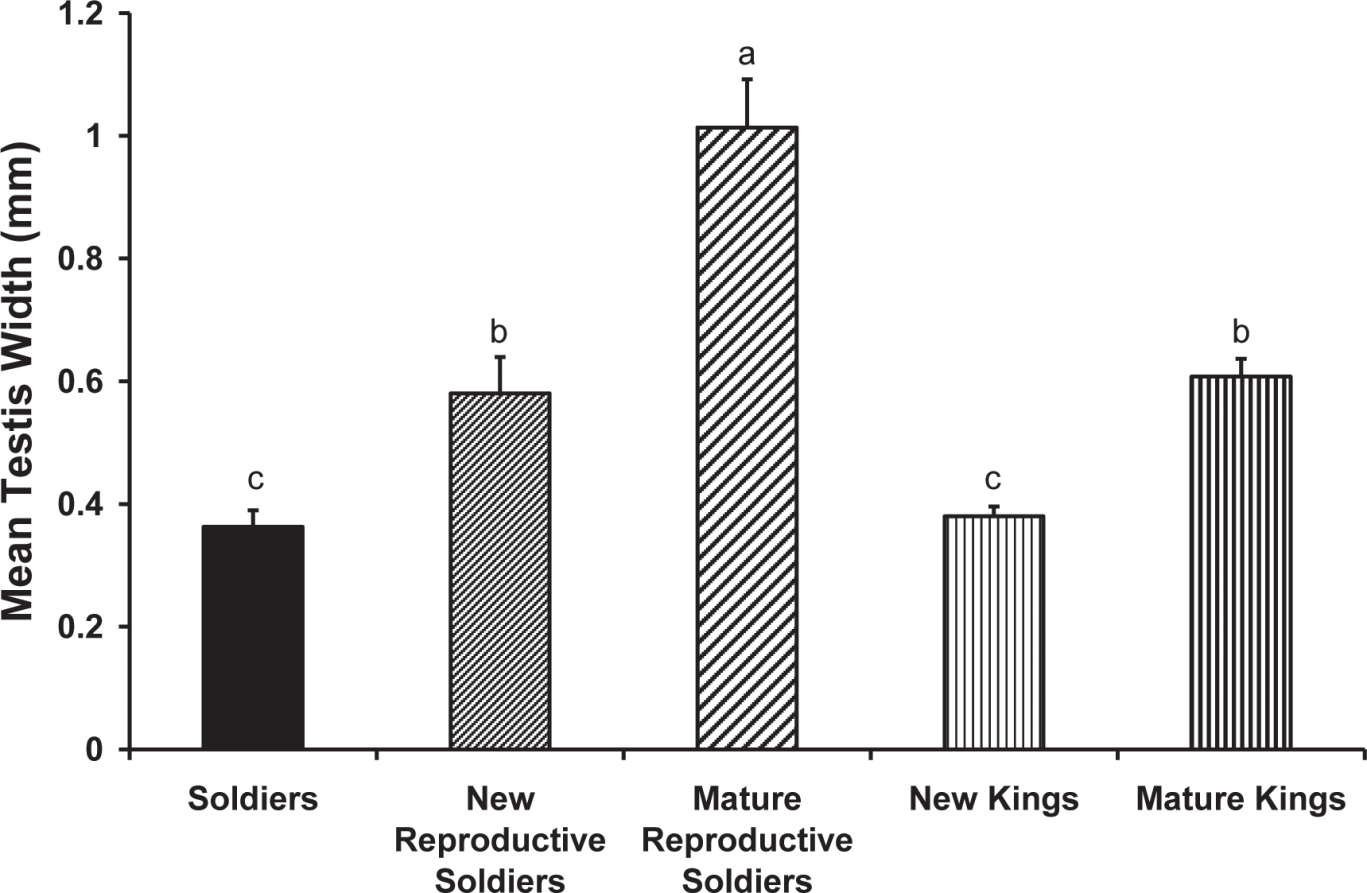
Comparison of means for testis width (mm) of male soldiers, reproductive soldiers and new and mature kings. Means with the same letter are not significantly different. Error bars indicate standard errors.

Testes (width) in recently eclosed reproductive soldiers were larger than mature sterile soldiers but smaller than mature reproductive soldiers (p < 0.0001). Dealate kings testes’ were equivalent in width to sterile soldiers’, while mature kings’ testes were equivalent to newly differentiated reproductive soldiers (Fig. 3).

***Correlation of live weight and testis width***

There was no correlation between live weight and testis width in sterile soldiers (p = 0.2952), new reproductive soldiers (p = 0.8225), mature reproductive soldiers (p = 0.0639) or new kings (p = 0.3071). Mature kings (n = 21) testis width and live weight correlated positively (p = 0.0448).

### Specimens of unknown age and live weight

***Morphological differences between soldiers and reproductive soldiers***

**Figure 4.**
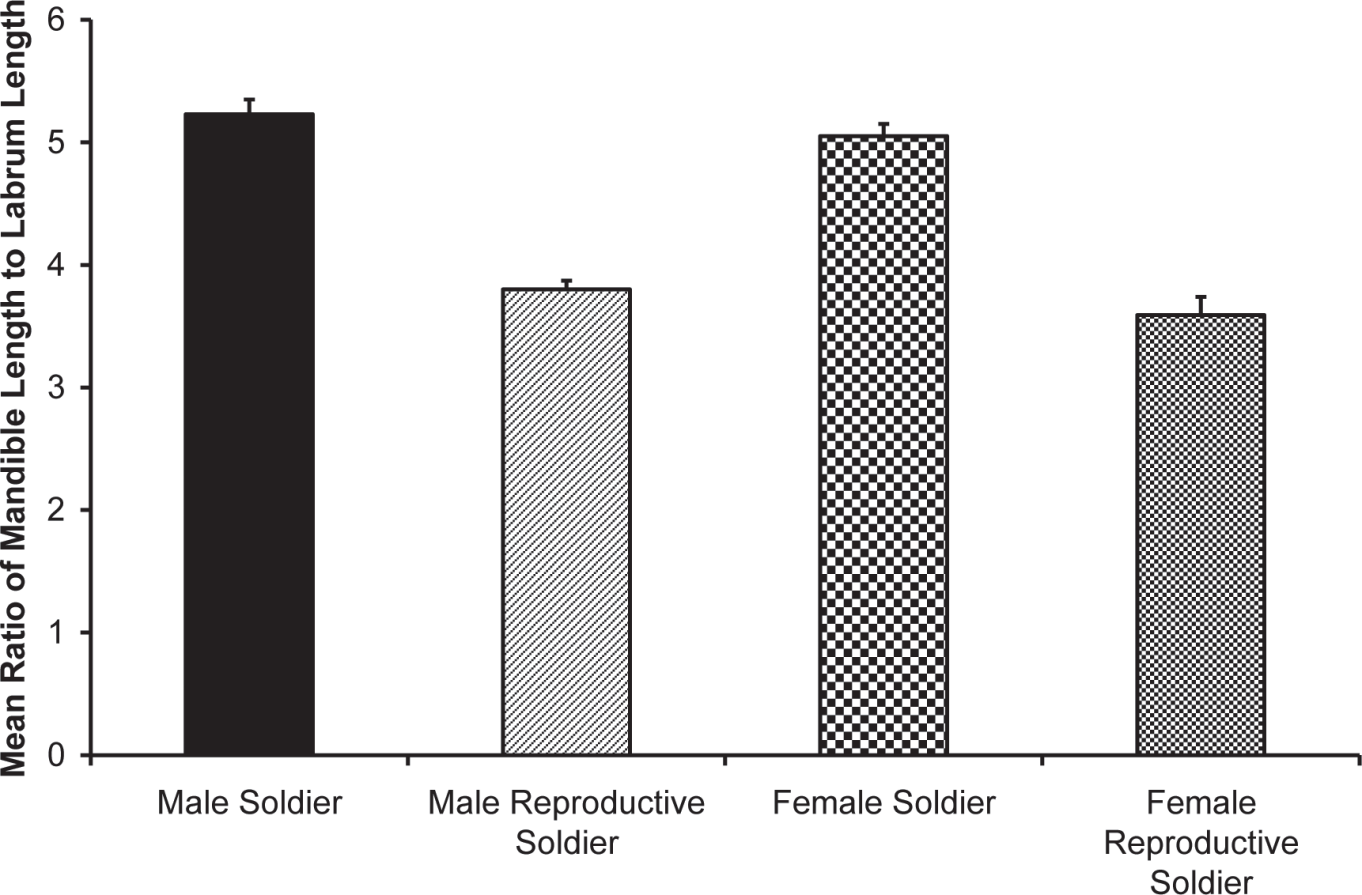
Comparison of means for ratio of mandible length to labrum length of male and female soldiers and reproductive soldiers. Within sex, soldiers had statistically significantly different mean ratios than reproductive soldiers (p < 0.01). Error bars indicate standard errors. Source data for the figure are listed in a table in the Supplementary Material.

Figure 4 shows pooled means by caste, after grouping male and female data because there was no significant sex effect. Morphological differences between soldiers and reproductive soldiers by sex are listed in Table 1. Multivariate ANOVA (MANOVA) for both male and female morphology indicated no overall caste by sex interaction (p > 0.4) and a significant overall caste effect (Wilks’ Lambda statistic, p < 0.01). (See supplementary material, Table 1, for comparison of means for significant quantitative measurements of castes using pooled male and female data with no significant sex or sex-by-caste interaction).

**Table 1.** Morphological differences between soldiers and reproductive soldiers by sex in *Zootermopsis n.nevadensis*

Variable	P-value of caste effect (males)		P-value of caste effect (females)	
	Mean (cm) ± SE, (n) [male soldiers]	Mean (cm) ± SE, (n) [male reproductive soldiers]	Mean (cm) ± SE, (n) [female soldiers]	Mean (cm) ± SE, (n) [female reproductive soldiers]
Eye Length	0.0002		0.2373	
	0.02 ± 0.0006 (84)	0.03 ± 0.0016 (38)	0.02 ± 0.0009 (60)	0.02 ± 0.002 (9)
Head Length	< 0.0001		< 0.0001	
	0.40 ± 0.0066 (84)	0.31 ± 0.0057 (38)	0.40 ± 0.0061 (58)	0.31 ± 0.010 (9)
Head Width	0.0004		0.0088	
	0.30 ± 0.0040 (84)	0.27 ± 0.0047 (38)	0.30 ± 0.0036 (60)	0.26 ± 0.0090 (9)
Labrum Length	0.7438		0.1484	
	0.06 ± 0.0009 (82)	0.06 ± 0.001 (38)	0.06 ± 0.0008 (60)	0.07 ± 0.002 (9)
Labrum Width	< 0.0001		< 0.0001	
	0.07 ± 0.0008 (82)	0.08 ± 0.001 (38)	0.07 ± 0.0008 (59)	0.08 ± 0.001 (9)
Mandible Length	<0.0001		< 0.0001	
	0.32 ± 0.0047 (82)	0.24 ± 0.0044 (38)	0.32 ± 0.0040 (60)	0.24 ± 0.010 (9)
Postmentum Length	< 0.0001		0.0009	
	0.28 ± 0.0055 (83)	0.20 ± 0.0053 (38)	0.28 ± 0.0062 (59)	0.21 ± 0.012 (9)
Postmentum Width (at widest point)	< 0.0001		0.0019	
	0.11 ± 0.0014 (83)	0.099 ± 0.0015 (38)	0.11 ± 0.0012 (60)	0.099 ± 0.0039 (9)
Postmentum Width (at narrowest point)	0.0010		0.1168	
	0.064 ± 0.00066 (83)	0.069 ± 0.0014 (38)	0.065 ± 0.00094 (60)	0.067 ± 0.0024 (9)
Ratio Mandible length: labrum length	< 0.0001		< 0.0001	
	5.23 ± 0.12 (81)	3.80 ± 0.072 (38)	5.05 ± 0.10 (60)	3.59 ± 0.15 (9)
Testes Diameter (smallest)	< 0.0001			
	0.033 ± 0.0012 (60)	0.074 ± 0.0036 (38)		
Testes Diameter (largest)	< 0.0001			
	0.042 ± 0.0015 (72)	0.094 ± 0.0050 (38)		
Ovary Length			< 0.0001	
			0.15 ± 0.0068 (53)	0.36 ± 0.051 (7)
Ovary Width (midpoint)			< 0.0001	
			0.019 ± 0.0013 (50)	0.070 ± 0.01 (7)
Ovary Width (widest point)			< 0.0001	
			0.025 ± 0.0013 (51)	0.087 ± 0.012 (7)

ANOVA results and descriptive statistics for each variable measured, by sex. Variables with p-values less than 0.05 were considered significant and are highlighted in bold. MANOVA indicated a significant overall caste effect for both males (p < 0.01) and females (p < 0.01) based on Wilks’ Lambda statistic.

Four of eight female reproductive soldiers had at least one egg; none of the 50 female normal soldiers examined had eggs. There was no significant difference between possession of wingbuds by caste.

***Correlations between external and internal morphology***

**Figure 5.**
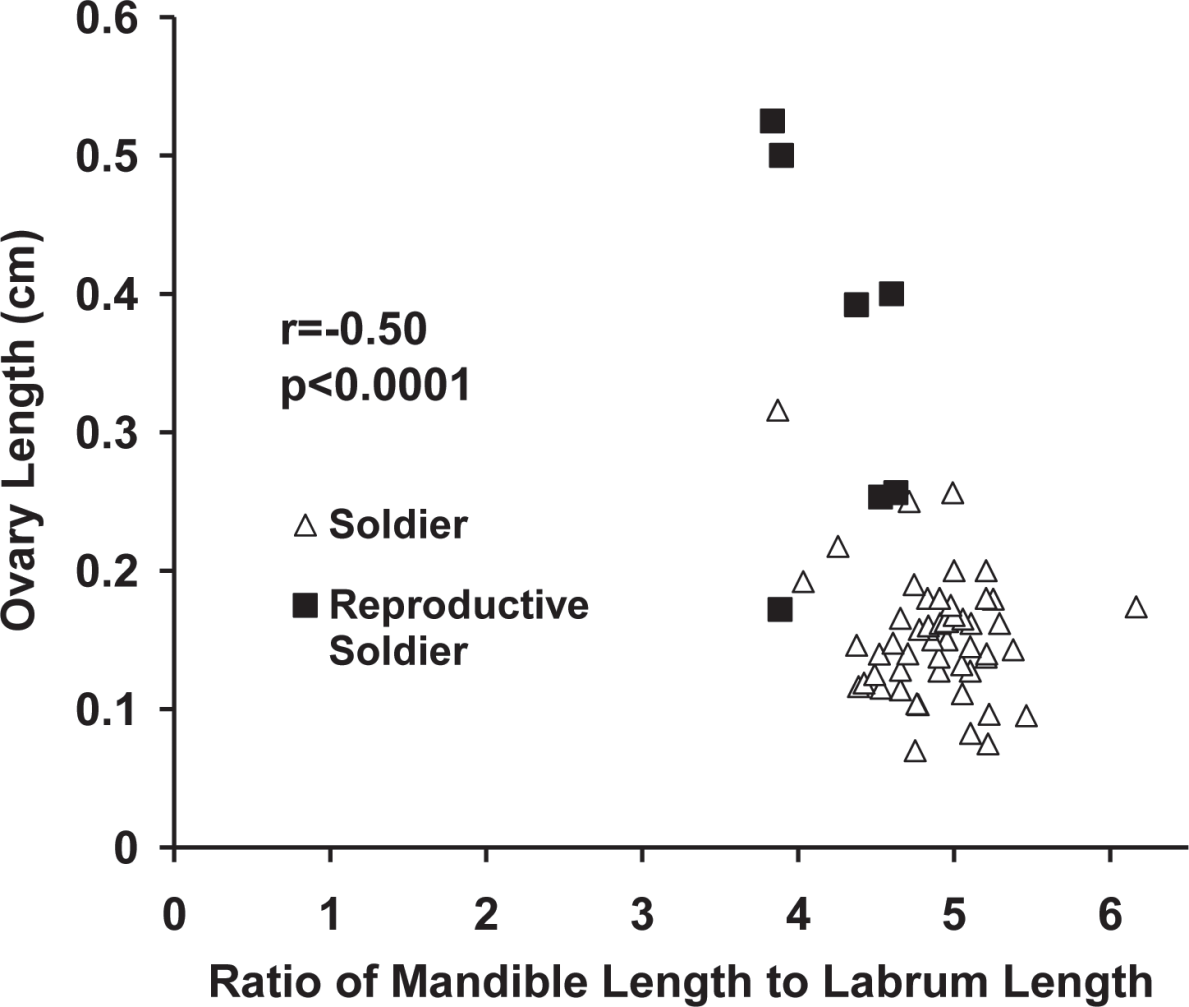
Correlation between ovary length and the ratio of mandible length to labrum length in female soldier morphs (soldiers and reproductive soldiers).

**Figure 6.**
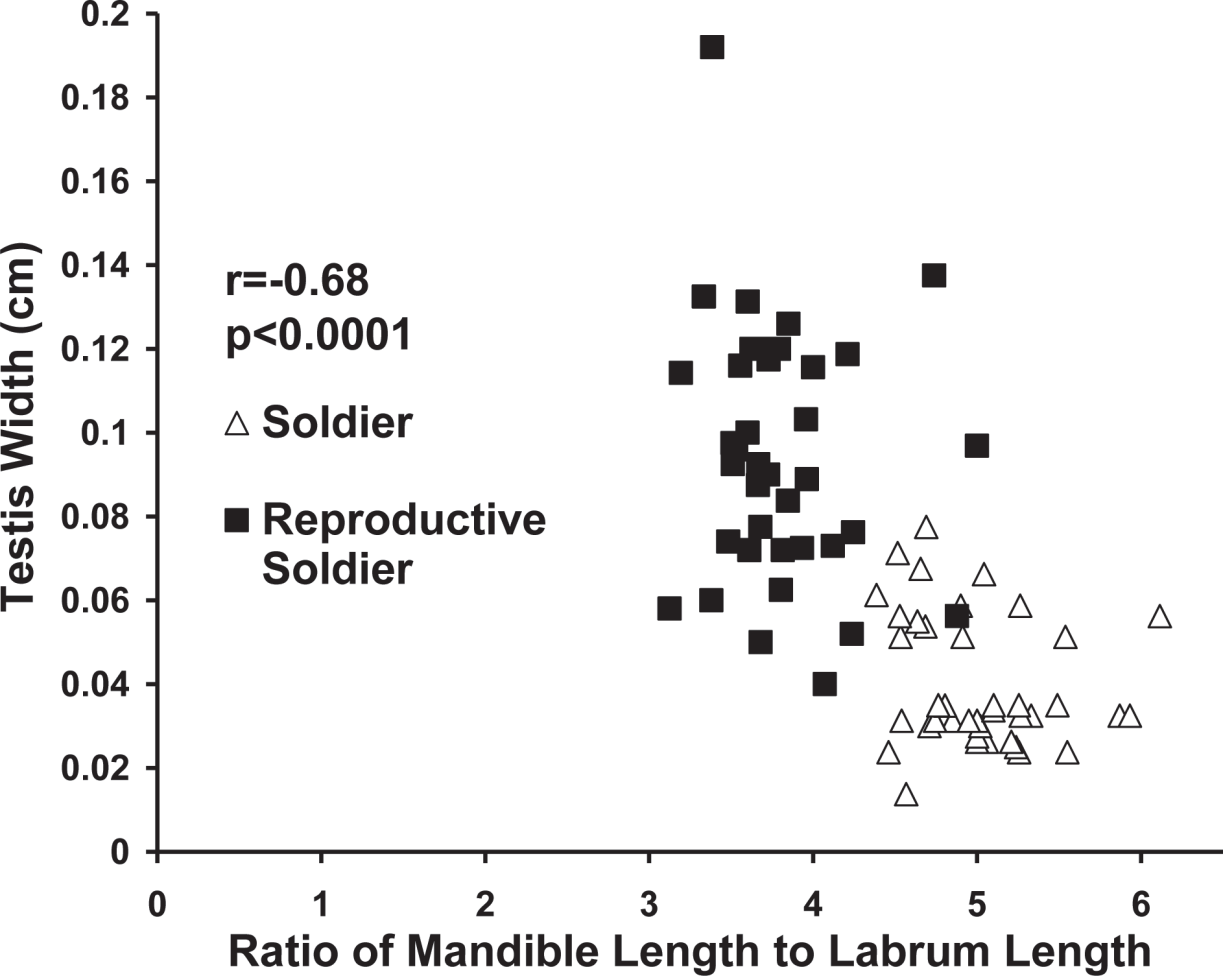
Correlation between testis diameter at the widest point and the ratio of mandible length to labrum length in male soldier morphs (soldiers and reproductive soldiers).

In female soldier morphs the ratio of mandible length to labrum length (Fig. 5) was correlated with ovary length. For male soldier morphs, the ratio of mandible length to labrum length (Fig. 6) was correlated with testis width. The lack of clear, discrete groups in Fig. 5, 6 was because newly differentiated RS were intermixed with developed RS in the archived material. Ovary length and testes width would have been much greater and the groups discrete following a few weeks of development. Figure 2 shows the progression and distinct separation of known age male soldiers and reproductive soldiers. (See Appendix I for the correlation table.)

***Homogeneity of gonad size variance between soldiers and reproductive soldiers***

Variance of ovary length was 0.0182 (mm^2^) in female reproductive soldiers which was much greater (p < 0.0001) than that for soldiers (0.000246) . Variance in ovary width at the widest point was also greater (p < 0.0001) in female reproductive soldiers (0.000106) than in normal soldiers (0.0000109). Variance in the widest diameter of the testis was greater (p < 0.0001) for male reproductive soldiers (0.0000955 mm^2^) than that for soldiers (0.0000231).

## Discussion

**Figure 7.**
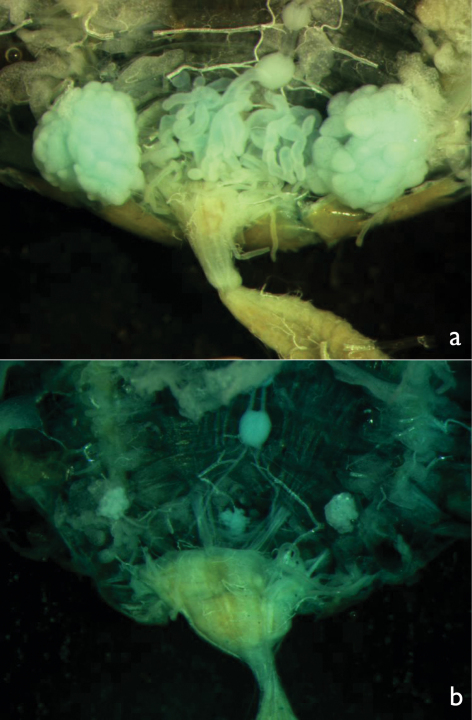
Testes and accessory glands of **a** male reproductive soldier and **b** male soldier. Both preparations were photographed under the same magnification.

**Figure 8.**
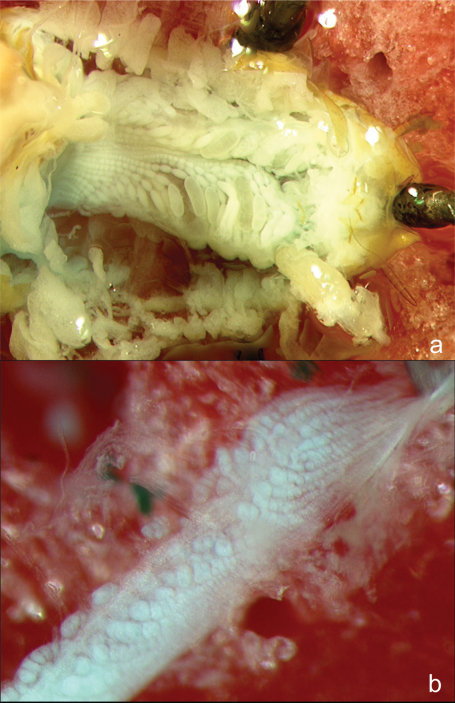
Ovaries of **a** a female reproductive soldier and **b** a female soldier. The female reproductive soldier ovarioles are much more developed and contain several eggs, while the female soldier ovarioles are reduced, with no evidence of egg development.

Neotenic soldiers of both sexes had smaller, rounder heads than soldiers (also observed although not formally analyzed by Heath 1928, Myles 1986), shorter (or more curved) mandibles, longer and more rectangular postmentums, wider and more oblong labrums, larger eyes, and a lower mandible-to-labrum ratio than normal/sterile soldiers. Reproductive soldiers of both sexes had larger gonads than soldiers. Male reproductive soldiers, whether newly eclosed or mature had larger testes than soldiers (Fig. 7). Unlike female reproductive soldiers, normal female soldiers never had developing eggs (Fig. 8). There was no difference between castes in terms of wingbud frequency. These results confirm the utility of these morphometrics to distinguish soldiers from reproductive soldiers. Due to age and size variance in reproductive soldiers, the morphometrics that involved ratios (head roundness, postmentum shape, labrum shape, and mandible-to-labrum ratio) are more reliable predictors of gonad development across a wide range of reproductive soldier sizes.

**Figure 9.**
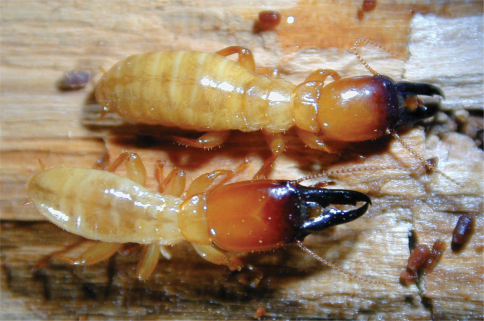
Male reproductive soldier (top) and male soldier (bottom). Note coloration of abdomen, shape of head capsule, and ratio of mandible to labrum length.

The external, ratio-based morphological measurement of mandible length to labrum length is a strong indicator of gonad size in soldier morphs of both sexes. This ratio accounts for body size and age differences and is visible before other characteristics (e.g. color and shape of abdomen) are apparent, serving as a useful, reliable correlate of gonad size in soldier morphs of a variety of ages. This ratio can easily be estimated—a labrum that extends less than a quarter of the length of the mandibles indicates a normal, sterile soldier. If the labrum is close to a third of the length of the mandible the individual is a reproductive soldier (Fig. 9).

Variation in gonad size may be due to age, as newly developed male reproductive soldiers had significantly smaller gonads than those that had been fertile for a longer period. Female reproductive soldiers had much greater variance in ovary length and ovary width at the widest point than did normal soldiers. This may be because of the inclusion of these younger, less sexually developed reproductive soldiers in the sample.

Morgan (1959) documented variation in head shape in the “emergency soldiers” of *Stolotermes ruficeps* Brauer, though he did not mention gonad development or reproductive status in these individuals. Similarly, Light (1943) noted a range of “intergrades” in *Zootermopsis nevadensis*, which he regarded as intercastes between soldiers, juveniles, nymphs, and neotenics. These observations suggest that individuals’ potential reproductive ability can be revealed without dissection or close observation of behavior. It should be noted that eggs were found only in females classified as reproductive soldiers. (Because sperm counts were not assessed, it is unknown whether all male “reproductive soldiers” were fertile.) The analysis of archived specimens could not distinguish between reproductive soldiers of varying sexual maturity; therefore, the continuum of morphology found in soldier morphs may in fact represent a continuum of reproductive soldier ages, as suggested by the wide variance found in reproductive soldier gonad sizes and data from known age individuals.

It is likely that modern reproductive soldiers represent an early step in soldier evolution, and that the loss of fertility in soldiers was secondary to the development of large mandibles and heavily sclerotized heads, advantageous for primitive termites in intercolony interactions (Thorne et al. 2003). The secondary loss of reproductive capacity after the evolution of soldier morphology also appears to have occurred in aphids (Stern and Foster 1997), thrips (Chapman et al. 2002) and ant soldiers (Urbani and Passera 1996), as reviewed by Thorne et al. (2003). Roisin (1999) suggested that reproductive soldiers’ distinctive morphology may merely be a non-adaptive accident of the dual roles of juvenile hormone as both a stimulus for soldier development as well as a gonadotropic hormone in reproductives. However, distinct roles and behaviors in meetings with neighboring colonies imply that this caste is not an accident (Thorne et al. 2003). Naturally occurring reproductive soldiers are distinct from artificially induced nymph/soldier intercastes. Miura et al. (2003) applied juvenile hormone analogue (JHA) to *Zootermopsis nevadensis* nymphs, causing them to molt into intercastes that share some characteristics with natural reproductive soldiers (e.g. small, round head; developed gonads; short, curved mandibles). The JHA induced intercastes had a range of morphologies, depending on the nymphal stage at which the JHA was applied. However, all possessed wings or wingbuds. Fully formed membranous wings have never been observed in a naturally occurring soldier or reproductive soldier.

Further study is needed to elucidate the developmental pathway of reproductive soldiers and to determine whether they result from a combination of developmental or social signals, or whether they develop in response to a single stimulus. Because reproductive soldiers are considered relictual transitional forms reflecting the evolutionary history of soldiers (Thorne et al. 2003) this work suggests that soldier development may have been much more flexible in the past than in most extant termites. Studies in progress will determine whether larger gonad size in mature reproductive soldiers compared to primary reproductives confers greater fertility and more rapid increase in colony size in a species with marked intraspecific competition for nesting resources.

## Appendix I

External morphology and gonad size: Complete means comparisons and correlations. (doi: 10.3897/zookeys.148.1672.app) File format: PDF

**Explanation note:** Table 1: Morphological differences between soldiers and soldier neotenics of *Zootermopsis nevadensis* and Table 2: Correlations between external morphology and gonad size in soldier morphs of *Zootermopsis nevadensis*

Copyright notice: This dataset is made available under the Open Database License (http://opendatacommons.org/licenses/odbl/1.0/). The Open Database License (ODbL) is a license agreement intended to allow users to freely share, modify, and use this Dataset while maintaining this same freedom for others, provided that the original source and author(s) are credited.
